# Correlation Between Pain Intensity and Trunk Sway in Seated Posture Among Office Workers with Chronic Spinal Pain: A Pilot Field-Based Study

**DOI:** 10.3390/s25051583

**Published:** 2025-03-05

**Authors:** Eduarda Oliosi, Afonso Caetano Júlio, Luís Silva, Phillip Probst, João Paulo Vilas-Boas, Ana Rita Pinheiro, Hugo Gamboa

**Affiliations:** 1 Laboratory for Instrumentation, Biomedical Engineering and Radiation Physics (LIBPhys), NOVA School of Science & Technology, NOVA University Lisbon, 2829-516 Caparica, Portugal; afonso.caetano93@gmail.com (A.C.J.); lmd.silva@fct.unl.pt (L.S.); p.probst@campus.fct.unl.pt (P.P.); hgamboa@fct.unl.pt (H.G.); 2 Research Centre in Physical Activity, Health, and Leisure (CIAFEL), Faculty of Sports, University of Porto, 4099-002 Porto, Portugal; 3 Centre for Research, Education, Innovation, and Intervention in Sport (CIFI2D) and Porto Biomechanics Laboratory (LABIOMEP), Faculty of Sports, University of Porto, 4200-450 Porto, Portugal; jpvb@fade.up.pt; 4 Institute of Biomedicine (iBiMED), School of Health Sciences, University of Aveiro, 3810-193 Aveiro, Portugal; anaritapinheiro@ua.pt

**Keywords:** musculoskeletal disorders, chronic pain, pain intensity, postural control, variability, inertial sensors

## Abstract

This pilot study examines the relationship between pain intensity and trunk sitting postural control in 10 office workers with chronic spinal pain, using field-based real-time inertial sensors. Pain intensity was assessed with the Numeric Pain Rating Scale (NPRS) before and after work across three non-consecutive workdays, while postural control was evaluated through estimated center of pressure (COP) displacements. Linear and nonlinear metrics, including sway range, velocity, the Hurst exponent, and sample entropy, were derived from the estimated COP time series. Pearson correlation coefficients (*r*) and corresponding *p*-values were used to analyze the relationship between pain intensity and postural control. Significant correlations, though limited to specific metrics, were found (*r* = −0.860 to 0.855; *p* < 0.05), suggesting that higher pain intensity may be correlated with reduced postural variability. These findings provide preliminary insights into the potential link between pain intensity and postural control. Understanding trunk posture dynamics could inform the development of targeted ergonomic interventions to reduce musculoskeletal stress and improve sitting comfort in office environments.

## 1. Introduction

Musculoskeletal disorders (MSDs), particularly chronic spinal conditions such as neck and lower back pain, represent a significant global occupational health challenge. By 2050, low back pain is projected to affect 843 million people, up from 619 million in 2020 [1], while neck pain, which impacted 203 million in 2020, is expected to rise to 269 million [2]. The increasing prevalence of sedentary office work, combined with aging populations and obesity rates, is expected to exacerbate this problem [1,3,4]. Office workers are especially vulnerable due to prolonged sitting, highlighting the urgent need for ergonomic strategies to mitigate the impacts of MSDs [5,6,7,8].

Addressing this issue requires innovative approaches, such as those proposed within the framework of Industry 4.0 and 5.0. These paradigms introduce opportunities to improve workplace health through technology integration and human-centered design. While Industry 4.0 emphasizes automation and digital transformation, Industry 5.0 prioritizes worker well-being and sustainable ergonomics [9,10,11]. In this context, addressing the health risks of prolonged sitting is essential. For example, nearly 40% of EU workers report sitting for excessive durations [5], prompting the EU-OSHA to recommend limiting sitting to less than half of the workday [5].

Movement variability and variation in posture represent complementary ergonomic principles that are particularly relevant in this regard. Movement variability refers to the natural, inherent fluctuations in motor performance across task repetitions [12]. This intrinsic property of biological systems [12,13] facilitates the redistribution of muscle activity, reducing localized fatigue, and improving activation patterns [14]. In contrast, variation in posture and movement involves intentional changes in body position, such as alternating between sitting and standing [15]. These postural adjustments are linked to enhancing comfort and productivity by up to 6.5% [16].

Recent reviews reinforce the benefits of these principles in workplace settings. Standing interventions have been shown to effectively reduce sedentary behavior without compromising productivity [17], while active breaks incorporating postural changes can alleviate pain and discomfort [18]. Advances in technologies, such as inertial measurement units (IMUs), further support ergonomic interventions by enabling the detailed analysis of movement and postural behaviors [19,20,21,22,23]. Together, these findings highlight the critical importance of movement variability as a central component of strategies to reduce back pain and improve workplace well-being.

Despite this growing recognition of the role of movement variability in reducing musculoskeletal discomfort, there remains a limited understanding of how specific aspects, such as trunk posture variability, influence pain intensity. Inconsistencies in kinematic data and a lack of focused studies hinder a comprehensive understanding of the role of movement in the management of chronic spinal pain [24,25].

This study aims to address these gaps by investigating the relationship between motor variability—measured through linear and nonlinear postural sway metrics derived from the estimated center of pressure (COP) time series—and pain intensity in office workers with chronic spinal pain, particularly those in tax authorities engaged in computer-based tasks while seated. By utilizing IMUs integrated into smartphones, this research seeks to advance our understanding of motor patterns and contribute to the development of interventions and digital health solutions tailored to this population. Based on the existing literature, the following is hypothesized:

1. Higher pain intensity is associated with reduced variability and complexity in trunk sway (e.g., lower entropy), reflecting increased trunk stiffness and a shift towards a more rigid and predictable postural control strategy. These adaptations likely serve as a compensatory mechanism to minimize movement-related stress and protect the spine during prolonged sitting [26,27,28,29].

2. Work-related activities modulate postural control and pain perception, leading to increased pain intensity and reduced movement variability in the post-work period (PM) compared to the pre-work period (AM). This effect may be attributed to cumulative biomechanical strain and fatigue associated with sustained "static" postures during prolonged occupational sitting [28,30].

## 2. Materials and Methods

### 2.1. Study Design

This cross-sectional study, part of the PrevOccupAI Project (Prevention of Occupational Disorders in Public Administrations using Artificial Intelligence), was conducted at offices of the Portuguese Tax and Customs Authority (AT) located in the Lisbon Metropolitan Area. A multidisciplinary team accomplished risk assessments for randomly selected tax enforcement professionals from the AT’s Human Resources department. As an incentive to participate in this study, evidence-based recommendations were developed to improve resilience and address occupational health challenges, informed by sources such as the ILO [31], the EU-OSHA [5], and Slater et al. [32]. Ethical approval was granted by NOVA University Lisbon (No. CE/FCT/005/2022) in accordance with the Declaration of Helsinki and GDPR. Informed consent was obtained from all participants.

### 2.2. Participants

A total of 10 workers were eligible to participate in this study. All participants were adults aged 18 years or older, with no history of neurological, orthopedic, rheumatic, oncological, or cardiorespiratory conditions; pregnant women were excluded. To meet the eligibility criteria, participants were required to have a history of non-specific spinal pain lasting at least three months, as defined by the International Association for the Study of Pain (IASP) and the International Classification of Diseases, 11th Revision (ICD-11) [33].

### 2.3. Procedures

A standardized data collection protocol was implemented using a dedicated cross-platform application developed within the PrevOccupAI project. This application enabled the acquisition of multimodal biosignals and self-reported questionnaire responses via smartphone and computer interfaces. Data collection spanned one workweek, adhering to protocols established in prior studies [34]. Each workday began with participants reporting to a designated workplace room for device setup. They first completed a daily pain questionnaire before the recording schedule was configured in the PrevOccupAI application. A smartphone, securely positioned on the chest, continuously recorded inertial sensor data and ambient noise throughout the workday, ensuring the uninterrupted monitoring of postural sway and movement patterns in a real-world occupational setting. Participants engaged in their regular work tasks while the smartphone passively captured movement-related data. At the end of the workday, participants returned for device removal and disinfection, followed by a second pain questionnaire to assess changes in pain perception. This methodology facilitated a comprehensive, ecologically valid evaluation of postural sway dynamics, leveraging smartphone-based sensing for continuous and unobtrusive monitoring.

#### 2.3.1. Demographics

Demographic data, including age, gender, height, and body mass, were captured using an integrated questionnaire module. The Body Mass Index (BMI) was calculated based on the standard formula BMI = mass (kg) / height (m)^2^. Data regarding work (years of work experience, weekly work schedule in hours) were also collected. Subjective assessments were conducted using validated instruments to assess physical activity levels, psychosocial risks, and chronic pain experiences.

#### 2.3.2. Pain Experience

In 2020, the IASP [35] revised its definition of pain to include both physical and emotional dimensions. Accordingly, pain perception was evaluated along three dimensions: intensity, distress, and interference [33].

Pain intensity was assessed daily using the Numerical Pain Rating Scale (NPRS), where participants reported their pain levels at the beginning and end of each workday, ranging from 0 (“no pain”) to 10 (“worst pain imaginable”). Pain-related distress, representing the emotional impact of persistent or recurrent pain, was measured weekly on an 11-point numerical scale, with 0 indicating “no distress” and 10 indicating “extreme pain-related distress”. Pain-related interference, quantifying the extent to which pain disrupted daily activities, was self-reported on a scale from 0 (no interference) to 10 (complete inability to perform activities) [33].

**Figure 1:**
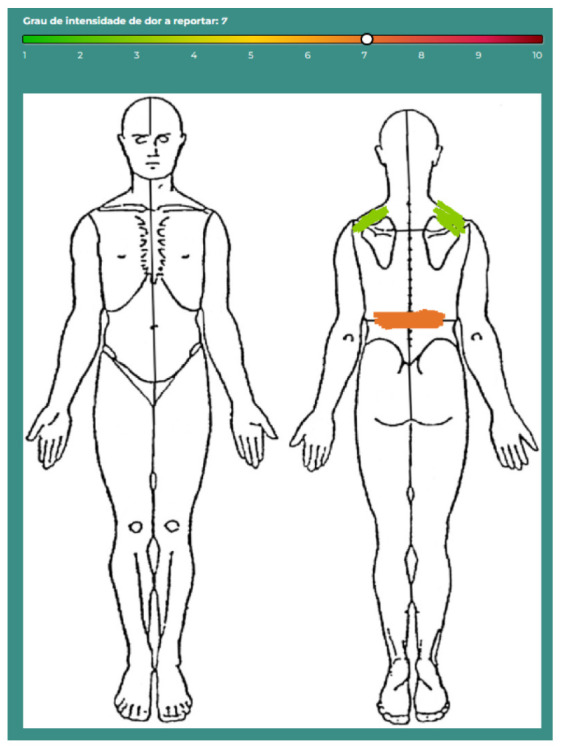
Interface for assessing pain location and intensity.

To optimize pain assessment, this project’s app integrated a body map tool, which allowed participants to precisely localize pain and quantify symptom distribution (Figure 1). Unmarked regions were assigned a pain intensity value of 0, ensuring a comprehensive and personalized representation of pain across anatomical regions.

#### 2.3.3. Physical Activity Levels

The European Portuguese version of the Short-Form International Physical Activity Questionnaire (IPAQ-SF) assessed physical activity, categorizing participants’ weekly activity as low, moderate, or high based on metabolic equivalents. This tool, validated for adults aged 18–65, captures vigorous, moderate, walking, and sitting activities over a past week [36].

#### 2.3.4. Psychosocial Risks

Workplace psychosocial risks were evaluated using the Portuguese version of the Copenhagen Psychosocial Questionnaire II (COPSOQ II) [37], covering multimodal domains such as work demands, interpersonal relations, and health and well-being [38]. This tool includes 76 items rated on a 5-point Likert scale. Results were grouped into their domains, expressed as percentages, and used to descriptively characterize the sample population, with input from occupational health stakeholders, management, and worker representatives [37].

#### 2.3.5. Data Collection and Analysis

**Figure 2:**
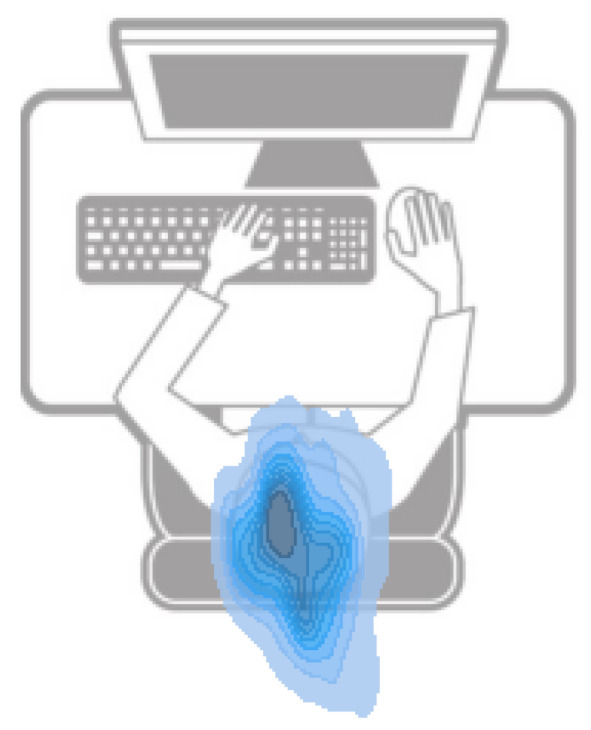
Illustration of COP sway during seated posture.

Data acquisition was performed using a Xiaomi Redmi Note 9 smartphone, which captured signals from the accelerometer (ACC), gyroscope (GYR), magnetometer (MAG), and rotation vector (RV). Sampling rates were constrained by the Android OS, with the ACC, GYR, and RV recorded at 100 Hz and MAG at 50 Hz. Motor biosignals were collected using these integrated sensors within the smartphone, which was securely fixed on the sternum with straps. The COP displacement time series represented an estimated COP derived from smartphone inertial sensor data. The collected data were represented in the orthogonal components of the anteroposterior (AP) and mediolateral (ML) directions, reflecting forward–backward (AP) and side-to-side (ML) trunk sway (see Figure 2). This approach provides a reliable method for quantifying postural sway, as supported by previous studies using force plates and smartphone-based inertial sensors to assess postural stability with demonstrated accuracy and consistency [39,40,41,42,43,44].

Linear measures of postural sway were computed from the COP time series. Key metrics included mean acceleration (in m/s^2^), standard deviation (SD) (in m/s^2^), sway range (in mm), sway area (in mm), sway path (in mm), and sway velocity (in mm/s). Sway range and area represent the maximum COP displacement in the resultant (overall) or AP and ML directions, sway path reflects the total COP distance traveled, and sway velocity indicates the rate of COP movement, offering insights into postural adjustment speed and variability [45,46,47]. These measures are indicators of centrality, describing magnitude and variability around a central point, and thus characterize movement quantity in data [48,49].

Nonlinear metrics, including Sample Entropy (SaEn) and Multifractal Detrended Fluctuation Analysis (MF-DFA), were used to assess postural control complexity (i.e., to describe the structure within the time series) [47]. SaEn quantifies sway regularity, where higher values indicate more complex and adaptable movement strategies. It also reflects how physiological health influences postural regulation by identifying signals under stationary conditions, such as reduced variability in less adaptable postural states. The Hurst exponent, derived from DFA, measures the long-term persistence and fractality of sway behavior. DFA analyzes time series by removing short-term fluctuations (detrending) to reveal long-term correlations, highlighting complex patterns in postural sway. DFA is assessed across different *q*-orders, which provide varying levels of detail: higher *q*-orders focus on fine-grained fluctuations, while lower *q*-orders capture broader trends. This multiscale analysis offers deeper insights into the dynamics and adaptability of postural control across different time scales [47,50,51,52,53].

The combination of these features was selected due to their widespread use in studies on seated posture, particularly in ergonomic and occupational contexts [54,55,56,57].

#### 2.3.6. Data Processing

All acquired signals were synchronized using timestamp alignment and resampled to a uniform frequency of 100 Hz to ensure consistency across modalities. The preprocessing pipeline for the ACC and RV data followed validated methodologies from previous studies [58]. The ACC data underwent a multi-step processing approach to remove noise and extract movement-related features. A low-pass filter with a 10 Hz cutoff was applied to suppress high-frequency noise and sensor artifacts. The gravitational acceleration component was then removed using an adaptive filtering technique to isolate dynamic movement. Systematic biases were corrected through detrending by subtracting the mean acceleration value from each sample, and a 150-sample moving average filter was used to smooth signal fluctuations. Similarly, RV signals were processed using a 5-sample moving average filter to minimize transient sensor noise and improve orientation stability. Given the extended duration of data collection (approximately 5 h per day), segmentation was performed using a 15-minute windowing approach to facilitate analysis. Data segments were classified into morning (AM1–AMx), lunch, and afternoon (PM1–PMx) periods.

To ensure that only seated postural sway data were analyzed, an algorithm was implemented to detect and exclude non-seated intervals based on acceleration magnitude thresholds. The magnitude of acceleration (mag) was computed as $mag=\sqrt{x_{\mathrm{acc}}^{2}+y_{\mathrm{acc}}^{2}+z_{\mathrm{acc}}^{2}}$, with a threshold of 2 m/s² derived from biomechanical analyses and metabolic equivalent (MET) calculations [59,60]. This threshold effectively distinguished seated from non-seated activities, ensuring that only relevant data were retained.

**Figure 3:**
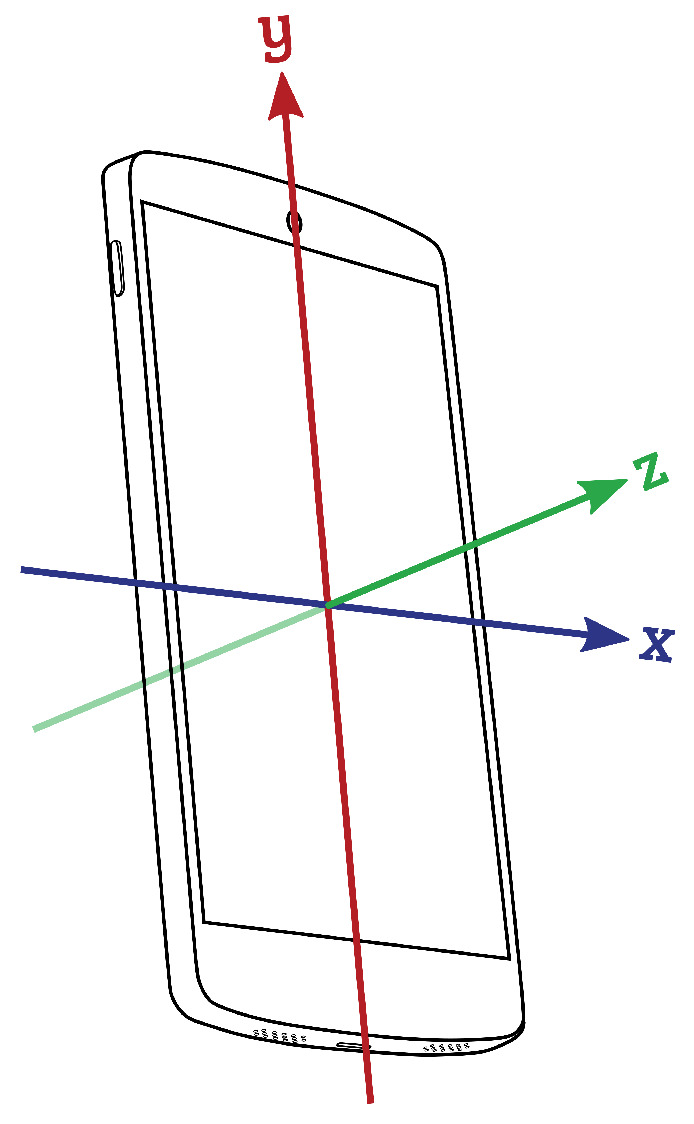
Coordinate system for smartphone-assisted postural analysis.

Postural sway was quantified by estimating the COP displacement from inertial sensor data. The RV signal was converted into quaternions and transformed into Euler angles to derive COP projections in the AP and ML directions. To ensure alignment across participants, the median of each Euler angle was subtracted to establish a reference position at the origin (0,0). The chest-mounted smartphone’s orientation was used to project Euler angles into the xz-plane, mapping postural movements throughout the workday (Figure 3). To standardize postural sway analysis, an elliptical boundary was applied based on prior research [61], with an AP radius of 25 mm and an ML radius of 18 mm. Data outside this predefined region were excluded to maintain consistency in postural sway assessment [58].

### 2.4. Previous Reporting on This Dataset

Previous analyses utilizing this dataset have examined how chronic spinal pain influences trunk movement patterns and postural dynamics in office settings. Ref. [58] applied a mixed ANOVA to analyze trunk sway, revealing that pain-free participants displayed distinct trunk movement characteristics in certain features compared to those with chronic spinal pain, particularly regarding fine motor adjustments. In another analysis, ref. [62] assessed postural variability among 40 office workers throughout the workday without segmenting by pain status or utilizing nonlinear metrics. The results indicated increased posture variability from morning to afternoon, with a notable rise in positional adjustments later in the day. This paper presents novel analyses focused on the relationship between postural dynamics and pain intensity, specifically within the cohort experiencing chronic spinal pain.

### 2.5. Statistical Analysis

Descriptive and inferential statistics were performed using SPSS 29. Sample demographics and questionnaire scores were used to characterize the participants. For continuous variables, normally distributed data were summarized with the mean and SD, while non-normally distributed data were described using the median and interquartile range (IQR). Categorical variables were reported as counts and proportions. Parametric assumptions were verified for normality using the Shapiro–Wilk test. To examine changes in pain intensity over time, Friedman’s Related-Samples Two-Way Analysis of Variance was applied. For correlations, Pearson’s (*r*) correlation coefficients were calculated to explore associations between sitting postural control dynamics (trunk displacement features in the AP and ML directions, along with resultant trunk sway time series) and perceived pain intensity (NPRS Sum of neck, upper back, and lower back pain). As Pearson’s *r* represents both the correlation coefficient and the effect size, it allows for a direct interpretation of the strength of relationships between variables. NPRS scores were summed across spinal regions to create an NPRS index. Summed pain intensity scores were analyzed for each day (Day 1: Monday; Day 3: Wednesday; Day 5: Friday) and period (AM: pre-work; PM: post-work). A significance threshold of α=0.05 was applied.

## 3. Results

The study sample consisted of 10 participants, 80% of whom identified as female. Physical activity levels, as assessed by the IPAQ-SF, indicated that 30% of participants engaged in low physical activity, 40% in moderate activity, and 30% in high activity levels. The mean age of the participants was 54 years (SD = 6.5). The average BMI was 26.75 kg/m² (SD = 6.14), with participants having an average of 18.2 years of work experience (SD = 14.06) and a typical weekly work schedule of approximately 40 h (SD = 4). On average, participants reported spending 9.4 h per day sitting (SD = 3.44), with a reduced average of 4.18 h of sitting on weekends (SD = 1.78). The characteristics of the participants are summarized in Table 1.

The COPSOQ II results indicated moderate job demands (67.5; SD = 2.7) and neutral perceptions of health and well-being (50.0; SD = 6.1). No offensive behavior was reported (0.0; IQR: 0–10). Social relations and leadership scored moderately (38.1; SD = 3.5), as did values’ alignment within the workplace (31.3; SD = 4.8) and the work–individual interface (42.6; SD = 11.8). Work organization and job content (41.7; SD = 4.6) also received moderate ratings.

### 3.1. Pain Experience

The distribution of reported pain locations among participants revealed that three (30.0%) experienced isolated neck pain, five (50.0%) reported a combination of neck and lower back pain, one (10.0%) had upper back pain, and one (10.0%) experienced pain in both the neck and upper back regions.

The disability levels, assessed using the 11-point NPRS, revealed the following median scores: neck disability, 2.00 (IQR: 0–4); upper back disability, 0.90 (IQR: 0–5); and lower back disability, 0.80 (IQR: 0–5). In terms of classification, 20% of participants reported no disability, 50% reported mild disability, and 30% reported moderate disability for neck pain. For upper back disability, 80% reported no disability, while 20% reported moderate disability. Regarding lower back disability, 70% reported no disability, 10% reported mild disability, and 20% reported moderate disability.

Regarding pain-related distress, also assessed using the NPRS, the median distress scores were as follows: neck distress, 2.50 (IQR: 0–6); upper back distress, 1.10 (IQR: 0–6); and lower back distress, 1.20 (IQR: 0–5). For neck distress, 10% of participants reported no distress, 70% reported mild distress, and 20% reported moderate distress. For upper back distress, 80% reported no distress, and 20% reported moderate distress. For lower back distress, 60% of participants reported no distress, 30% reported mild distress, and 10% reported moderate distress.

Regarding pain intensity, which was assessed both before and after work each day, a detailed description can be found in Table 2. No statistically significant differences were found across days, periods, and pain locations (*p* > 0.05).

### 3.2. Correlations

Daily pain intensity (NPRS Sum) analysis across six assessment points (Day 1 AM/PM, Day 3 AM/PM, Day 5 AM/PM) revealed occasional correlations with postural sway metrics. While most correlations did not reach statistical significance (p>0.05), several moderate-to-strong correlations were observed, indicating potential relationships that merit further investigation. A summary of significant correlations between the NPRS and trunk sway metrics is provided in Table 3, with comprehensive correlation matrices for both linear and nonlinear sway features available in Appendix A.

For linear measurements (Table A1, Table A2, Table A3, Table A4, Table A5 and Table A6), significant correlations with the NPRS Sum were observed at different time points. On Day 1 in the morning, the NPRS Sum had a significant negative correlation with overall sway range (r=−0.688;p<0.05). In contrast, on Day 1 in the afternoon, the NPRS Sum exhibited a positive correlation with overall sway range (r=0.750;p<0.05) and with range in the AP direction (r=0.855;p<0.01). Additionally, on Day 5 in the morning, a strong negative correlation between the NPRS Sum and the standard deviation in the AP direction (SD AP) was noted (r=−0.719;p<0.05).

Nonlinear sway parameters showed varied correlations with the NPRS Sum across time points (Table A7, Table A8, Table A9, Table A10, Table A11 and Table A12). On Day 3 in the AM, several strong correlations were observed. The Hurst exponents at the scale H(0) in both the AP (r=−0.812;p<0.01) and ML (r=−0.860;p<0.01) directions, as well as at the scale H(4.5) in the AP (r=−0.786;p<0.01) and ML (r=−0.780;p<0.01) directions, were significantly negatively correlated with the NPRS Sum. SaEn in the ML direction also exhibited a significant negative correlation with the NPRS Sum (r=−0.703;p<0.05). On Day 3 in the PM, no significant correlations were found between the NPRS Sum and the nonlinear sway parameters. However, on Day 5 in the PM, the NPRS Sum showed a negative correlation with the Hurst exponent at the scale H(2) in the AP direction (r=−0.722;p<0.05).

Furthermore, moderate to strong positive intercorrelations were found among both linear and nonlinear postural sway parameters (Appendix A).

## 4. Discussion

This study investigated the relationship between pain intensity, measured using the NPRS Sum for neck, upper, and lower back pain, and postural sway characteristics in office workers with chronic spinal pain. IMUs were employed for real-time, field-based posture assessment. Both linear and nonlinear sway metrics were analyzed during pre- and post-work periods on Days 1 (Monday), 3 (Wednesday), and 5 (Friday) to assess their potential as objective indicators of pain intensity within occupational settings.

The findings revealed limited and variable correlations between pain intensity and postural sway metrics across different time points, illustrating the intrinsic intricacy of postural control systems in the context of chronic pain.

### 4.1. Interpretation of Results

Linear metrics, including range (in mm), mean acceleration (in m/s^2^), SD (in m/s^2^), velocity (in mm/s), sway path length (in mm), and sway path area (in mm^2^), were used to assess postural control. Nonlinear techniques, MF-DFA and SaEn, were applied to capture the postural control complexity, reflecting subtle changes that linear metrics might have missed.

Variations in correlations among linear metrics highlighted the nuances of seated postural dynamics. On Day 1, reduced morning sway variability (negative correlation with overall sway range) indicated more rigid postural control, while increased afternoon fluctuations (positive correlations with overall sway range and AP range) suggested compensatory adjustments. By Day 5, greater AP sway variability (negative correlation with SD) was linked to higher pain intensity. These findings are consistent with previous studies. For instance, Søndergaard et al. [54], reported positive correlations between discomfort and the standard deviations of COP displacement in both the AP and ML directions, alongside negative correlations with SaEn. This suggests that increased sway variability and reduced postural control complexity during seated tasks can be associated with greater perceived discomfort. Similarly, Madeleine et al. [63] observed that prolonged sitting led to a greater SD and lower SaEn in COP signals, reflecting decreased postural complexity and increased discomfort. Overall, these findings highlight the link between postural variability degradation and increased discomfort, reflecting the interplay between adaptive posture control and pain perception.

Correlations between nonlinear metrics and pain intensity varied notably across time points. No significant correlations were observed on Day 1 (AM and PM), Day 3 in the PM, or Day 5 in the AM. However, on Day 3 in the AM, strong negative correlations were observed for H(0) (AP and ML), H(4.5) (AP and ML), and SaEn (ML), suggesting a decline in postural sway complexity. On Day 5 in the PM, a significant negative correlation was also found for H(2) in the AP direction, indicating reduced postural adaptability. This reduction in variability aligns with the concept of a more periodic, less adaptive postural strategy, characterized by the loss of multiscale fractal complexity under pathological conditions [12,64]. These findings suggest that higher pain intensity is correlated with diminished variability at both micro-scale fluctuations (H(0)) and larger sway deviations (H(4.5)), as well as reduced entropy (SaEn), which reflect a shift toward more rigid, less flexible postural control [12]. The absence of consistent correlations across other periods underscores the dynamic and context-dependent nature of postural regulation in response to pain, consistent with theories of adaptive control in motor behavior [27].

Although most postural sway parameters showed weak correlations with the NPRS Sum, specific linear and nonlinear metrics demonstrated moderate to strong correlations at particular time points, indicating pain-related changes in postural stability. Hypothesis 2, predicting stronger evening (PM) correlations, was not supported. Overall, our results suggest a weak link between pain intensity and postural sway dynamics, likely due to the multifactorial nature of chronic pain, influenced by biopsychosocial factors [26,65,66]. Furthermore, variability in sitting habits and postural adjustments also contributes to inconsistent sway patterns (e.g., “breakers” vs. “prolongers”) [56].

Our findings highlight the potential for the development of personalized ergonomic interventions aimed at protecting office workers from prolonged exposure to postural stress and pain. By understanding trunk posture dynamics, tailored strategies can help reduce MSDs and improve comfort, as optimal postures vary between individuals [32].

### 4.2. Limitations and Future Research

Several limitations should be considered. Data were collected from an adult population with an average age of 54 years (SD = 6.5), while previous studies emphasize age-related differences in variability [49,67], particularly in office workers [55]. Thus, the kinematic measurements in this study may not reflect those of other age groups or occupations. The use of smartphone IMUs, while practical, introduced limitations, as placement variability and daily reapplication increased the risk of random errors in postural sway measurements due to inconsistent placement [68], despite the use of standardized straps and consistent researcher handling.

As this study was conducted in a field setting, variations in cognitive load (e.g., focused computer work) may have influenced postural sway. Prior research suggests that cognitive dual-tasking can reduce postural sway in chronic low back pain, though effects are more pronounced in complex balance tasks [69]. Similar effects were observed in healthy adults, where cognitive demands altered postural complexity without significantly affecting displacement measures [70]. Cognitive task difficulty has also been linked to changes in postural variability, particularly in children and older adults, who demonstrate increased sway area and complexity under more challenging conditions [71]. Future studies should integrate controlled cognitive-load assessments to better isolate the effects of attentional demands on postural control, such as using EEG [72].

Customer service roles in public administration and finance face substantial psychosocial risks. For example, 31% of EU workers report suppressing emotions due to customer anger and abuse [73]. In tax offices, depression has been linked to high trait anxiety, workplace conflicts, and low job satisfaction, making tax workers particularly vulnerable to MSDs [74,75,76,77]. These psychosocial hazards limit the applicability of the findings to other office roles, such as programmers and call center staff. However, the non-alarming COPSOQ II results in this study suggest a lower psychosocial risk in the analyzed sample.

While our findings suggest a weak correlation between pain intensity and postural sway metrics, they underscore the complexity of chronic pain management. This highlights the need for individualized, multidimensional approaches to postural assessment and ergonomic interventions, where pain intensity is considered alongside other psychosocial and physical factors. Future interventions may benefit from a combination of sensor data and self-reported measures to provide more accurate assessments of pain and postural dynamics, guiding more effective ergonomic strategies in the workplace.

Although a one-week observation period was chosen to align with this study’s primary objectives, future research could benefit from longer observation durations to capture greater variability in postural dynamics and explore potential long-term trends. Additionally, while the small sample size limits the generalizability of the findings, future studies with larger, more diverse samples will offer more robust and conclusive insights. Given the multiple comparisons conducted in this study, the potential for Type I errors must be considered. Future research should apply corrections to account for multiple tests, thereby enhancing the robustness of the findings.

## 5. Conclusions

Although postural sway parameters showed weak correlations with pain intensity, specific metrics revealed stronger correlations, suggesting potential links between pain and postural stability. These findings highlight the need for more research on motor variability and pain intensity to inform ergonomic interventions. While our findings were not strongly significant, they contribute to assessing postural sway in real-world settings and provide exploratory insights into how movement-based metrics may inform pain assessment, ergonomic interventions, and future rehabilitation strategies for individuals with chronic spinal pain.

Hypothesis 1 was partially supported, with pain intensity correlated with the reduced complexity of postural sway at specific points, although the effects were inconsistent. Hypothesis 2 was not supported, as work activities did not consistently increase post-work pain or reduce variability, suggesting that other factors may influence postural control and pain intensity. Future research should involve larger sample sizes, longitudinal designs, randomized controlled trials, and consider pain location to better explore these relationships. Furthermore, utilizing a full range of digital health resources, including wearable sensors, could provide valuable information on postural dynamics and pain management.

## Figures and Tables

**Table 1:** Sample characterization.

Variable	Outcome
Gender	80% Female (8 Females, 2 Males)
Physical Activity Levels	Low: 30%; Moderate: 40%; High: 30%
Mean Age (years)	54 ± 6.5
Mean BMI (kg/m²)	26.75 ± 6.14
Work Experience (years)	18.2 ± 14.06
Weekly Work (h)	40 ± 4
Daily Sitting Time (h)	9.4 ± 3.44
Weekend Sitting Time (h)	4.18 ± 1.78

**Table 2:** Pain intensity scores based on the NPRS.

Variable	AM			PM		
	Median	IQR	95% CI	Median	IQR	95% CI
Day 1						
Neck	0.00	4	(−0.26, 3.06)	0.00	2	(−0.61, 3.01)
Upper Back	0.00	1	(−0.47, 2.27)	0.00	1	(−0.55, 2.35)
Lower Back	0.00	5	(0.12, 3.88)	0.00	4	(−0.29, 3.09)
Day 3						
Neck	0.00	3	(−0.27, 2.47)	0.00	3	(−0.22, 2.62)
Upper Back	0.00	0	(−0.63, 1.63)	0.00	1	(−0.55, 2.35)
Lower Back	0.00	0	(0.00, 0.00)	0.00	4	(−0.29, 3.09)
Day 5						
Neck	0.00	3	(−0.23, 2.43)	0.00	3	(−0.30, 2.70)
Upper Back	0.00	1	(−0.37, 1.77)	0.00	5	(−0.02, 3.82)
Lower Back	0.00	3	(−0.17, 2.17)	0.00	4	(−0.09, 3.49)
NPRS Sum						
Day 1	4.0	9	(1.10, 7.50)	2.5	6	(0.54, 6.46)
Day 3	0.0	3	(−0.72, 3.92)	3.5	5	(0.76, 6.24)
Day 5	0.0	6	(−0.32, 5.92)	3.5	8	(0.51, 9.09)

AM: pre-work; PM: post-work; NPRS: Numeric Pain Rating Scale; IQR: interquartile range; CI: Confidence Interval.

**Table 3:** Significant correlations between NPRS Sum and linear/nonlinear metrics.

	Day	Period	Metric	Direction	Correlation
NPRS vs.	1	AM	Overall range	Negative	−0.688 *
	1	PM	AP range	Positive	0.855 **
	1	PM	Overall range	Positive	0.750 *
	3	AM	H(0) AP	Negative	−0.812 **
	3	AM	H(0) ML	Negative	−0.860 **
	3	AM	H(4.5) AP	Negative	−0.786 **
	3	AM	H(4.5) ML	Negative	−0.780 **
	3	AM	SaEn ML	Negative	−0.703 *
	5	AM	SD AP	Negative	−0.719 *
	5	PM	H(2)	Negative	−0.722 *

Abbreviations: NPRS: Numeric Pain Rating Scale; SD: standard deviation; AP: anteroposterior; ML: mediolateral; H: Hurst exponent. Significance levels: * *p* < 0.05; ** *p* < 0.01.

## Data Availability

The data presented in this study are available upon reasonable request from the corresponding author.

## Appendix A

The following tables present the correlation matrices for various sway metrics and pain intensity. Table A1, Table A2, Table A3, Table A4, Table A5 and Table A6 summarize the linear correlations between sway metrics and pain intensity across different days and time periods. Table A7, Table A8, Table A9, Table A10, Table A11 and Table A12 provide the nonlinear correlation matrices for the same metrics and conditions.

**Table A1:** Correlation Matrix of Linear Measurements on Day 1 During the AM Period and NPRS Sum (Day 1 AM).

	Mean		SD		Range			Area	Path	Velocity
	AP	ML	AP	ML	AP	ML	Overall			
NPRS Sum	−0.064	−0.348	0.351	0.041	−0.592	−0.461	−0.688 *	−0.552	−0.594	−0.593
Mean AP		0.449	−0.174	−0.013	−0.119	−0.424	−0.253	−0.226	−0.070	−0.073
Mean ML			−0.662 *	−0.099	0.339	−0.112	0.221	0.071	0.057	0.033
SD AP				0.662 *	−0.457	0.257	−0.241	−0.529	−0.383	−0.371
SD ML					−0.328	0.241	−0.168	−0.651 *	−0.580	−0.582
Range AP						0.173	0.904 **	0.612	0.547	0.547
Range ML							0.571	0.475	0.552	0.545
Range Overall								0.706 *	0.700 *	0.698 *
Area									0.956 **	0.954 **
Path										0.999 **

AM: Pre-work; NPRS: Numeric Pain Rating Scale; SD: Standard Deviation; AP: Anteroposterior; ML: Mediolateral; *: p<0.05; **: p<0.01.

**Table A2:** Correlation Matrix of Linear Measurements on Day 1 During the PM Period and NPRS Sum (Day 1 PM).

	Mean		SD		Range			Area	Path	Velocity
	AP	ML	AP	ML	AP	ML	Overall			
NPRS Sum	0.007	0.199	−0.196	−0.600	0.855 **	0.143	0.750 *	0.345	0.378	0.393
Mean AP		−0.245	0.152	−0.040	−0.061	−0.138	−0.108	−0.318	−0.304	−0.306
Mean ML			0.322	−0.387	−0.026	−0.688 *	−0.234	0.014	−0.106	−0.053
SD AP				0.247	0.032	−0.220	−0.055	−0.118	−0.212	−0.196
SD ML					−0.341	0.377	−0.174	0.150	0.169	0.171
Range AP						0.467	0.965 **	0.582	0.596	0.594
Range ML							0.678 *	0.628	0.718 *	0.678 *
Range Overall								0.660 *	0.698 *	0.685 *
Area									0.984 **	0.987 **
Path										0.997 **

PM: Post-work; NPRS: Numeric Pain Rating Scale; SD: Standard Deviation; AP: Anteroposterior; ML: Mediolateral; *: p<0.05; **: p<0.01.

**Table A3:** Correlation Matrix of Linear Measurements on Day 3 During the AM Period and NPRS Sum (Day 3 AM).

	Mean		SD		Range			Area	Path	Velocity
	AP	ML	AP	ML	AP	ML	Overall			
NPRS Sum	0.148	0.456	0.095	−0.127	−0.006	−0.368	−0.163	−0.047	0.085	0.059
Mean AP		−0.278	−0.447	−0.449	0.039	−0.068	−0.008	−0.306	−0.074	−0.077
Mean ML			0.375	−0.066	−0.072	−0.497	−0.256	0.211	0.009	−0.017
SD AP				0.765 **	−0.377	−0.382	−0.403	−0.136	−0.305	−0.310
SD ML					−0.333	−0.082	−0.240	−0.170	−0.323	−0.313
Range AP						0.695 *	0.947 **	0.743 *	0.914 **	0.915 **
Range ML							0.888 **	0.550	0.720 *	0.732 *
Range Overall								0.721*	0.905 **	0.911 **
Area									0.887 **	0.889 **
Path										0.999 **

AM: Pre-work; NPRS: Numeric Pain Rating Scale; SD: Standard Deviation; AP: Anteroposterior; ML: Mediolateral; *: p<0.05; **: p<0.01.

**Table A4:** Correlation Matrix of Linear Measurements on Day 3 During the PM Period and NPRS Sum (Day 3 PM).

	Mean		SD		Range			Area	Path	Velocity
	AP	ML	AP	ML	AP	ML	Overall			
NPRS Sum	0.505	−0.366	−0.011	−0.370	0.573	0.210	0.478	0.214	0.297	0.269
Mean AP		−0.504	0.049	0.031	−0.194	−0.193	−0.236	−0.180	−0.291	−0.279
Mean ML			0.316	0.164	0.067	0.200	0.164	−0.199	−0.088	−0.101
SD AP				0.495	−0.223	0.298	0.026	−0.417	−0.362	−0.373
SD ML					−0.282	0.491	0.024	−0.128	−0.188	−0.139
Range AP						0.588	0.926 **	0.674 *	0.842 **	0.829 **
Range ML							0.843 **	0.433	0.556	0.580
Range Overall								0.591	0.777 **	0.734 **
Area									0.969 **	0.974 **
Path										0.996 **

PM: Post-work; NPRS: Numeric Pain Rating Scale; SD: Standard Deviation; AP: Anteroposterior; ML: Mediolateral; *: p<0.05; **: p<0.01.

**Table A5:** Correlation Matrix of Linear Measurements on Day 5 During the AM Period and NPRS Sum (Day 5 AM).

	Mean		SD		Range			Area	Path	Velocity
	AP	ML	AP	ML	AP	ML	Overall			
NPRS Sum	0.288	−0.085	−0.719 *	−0.054	−0.134	−0.082	−0.112	−0.247	−0.165	−0.173
Mean AP		−0.286	−0.467	−0.115	0.096	−0.218	0.025	0.086	−0.190	−0.191
Mean ML			0.217	−0.449	−0.295	−0.367	−0.351	−0.291	−0.230	−0.219
SD AP				0.506	0.457	0.363	0.435	0.378	0.393	0.410
SD ML					0.546	0.687 *	0.642 *	0.507	0.568	0.579
Range AP						0.637 *	0.957 **	0.855 **	0.743 *	0.748 *
Range ML							0.826 **	0.730 *	0.907 **	0.908 **
Range Overall								0.912 **	0.878 **	0.883 **
Area									0.879 **	0.885 **
Path										0.999 **

AM: Pre-work; NPRS: Numeric Pain Rating Scale; SD: Standard Deviation; AP: Anteroposterior; ML: Mediolateral; *: p<0.05; **: p<0.01.

**Table A6:** Correlation Matrix of Linear Measurements on Day 5 During the PM Period and NPRS Sum (Day 5 PM).

	Mean		SD		Range			Area	Path	Velocity
	AP	ML	AP	ML	AP	ML	Overall			
NPRS Sum	−0.229	−0.501	−0.379	−0.391	0.367	0.493	0.429	0.211	0.201	0.182
Mean AP		0.416	0.233	−0.090	−0.254	0.067	−0.162	−0.060	−0.180	−0.197
Mean ML			0.327	0.405	−0.691 *	−0.677 *	−0.717 *	−0.489	−0.501	−0.497
SD AP				−0.116	0.250	0.024	0.182	0.417	0.377	0.397
SD ML					−0.669 *	−0.642 *	−0.680 *	−0.664 *	−0.539	−0.532
Range AP						0.842 **	0.983 **	0.951 **	0.944 **	0.943 **
Range ML							0.926 **	0.759 *	0.806 **	0.775 **
Range Overall								0.918 **	0.929 **	0.916 **
Area									0.941 **	0.944 **
Path										0.996 **

PM: Post-work; NPRS: Numeric Pain Rating Scale; H: Hurst Exponent; SaEn: Sample Entropy; AP: Anteroposterior; ML: Mediolateral; *: p<0.05; **: p<0.01.

**Table A7:** Correlation Matrix of Nonlinear Measurements on Day 1 During the AM Period and NPRS Sum (Day 1 AM).

	H(−5)		H(0)		H(2)		H(4.5)		SaEn	
	AP	ML	AP	ML	AP	ML	AP	ML	AP	ML
NPRS Sum	0.255	−0.100	0.212	−0.107	−0.300	−0.403	0.098	−0.273	0.263	0.378
H(−5) AP		−0.079	0.221	0.116	0.058	−0.218	0.460	−0.120	−0.061	0.057
H(−5) ML			0.462	0.615	0.712 *	0.486	0.220	0.261	0.211	0.016
H(0) AP				0.231	0.625	0.057	0.555	−0.084	−0.388	−0.065
H(0) ML					0.654 *	0.862 **	0.335	0.787 **	−0.029	−0.486
H(2) AP						0.655 *	0.718 *	0.391	−0.487	−0.513
H(2) ML							0.212	0.864 **	−0.260	−0.750 *
H(4.5) AP								0.072	−0.606	−0.397
H(4.5) ML									−0.083	−0.752 *
SaEn AP										0.671 *

AM: Pre-work; NPRS: Numeric Pain Rating Scale; H: Hurst Exponent; SaEn: Sample Entropy; AP: Anteroposterior; ML: Mediolateral; *: p<0.05; **: p<0.01.

**Table A8:** Correlation Matrix of Nonlinear Measurements on Day 1 During the PM Period and NPRS Sum (Day 1 PM).

	H(−5)		H(0)		H(2)		H(4.5)		SaEn	
	AP	ML	AP	ML	AP	ML	AP	ML	AP	ML
NPRS Sum	−0.075	0.278	−0.257	0.153	−0.112	−0.236	−0.366	−0.100	−0.372	−0.437
H(−5) AP		0.462	0.533	0.524	0.671 *	−0.044	0.648 *	−0.090	0.146	−0.201
H(−5) ML			0.338	0.733 *	0.252	0.204	0.463	0.241	−0.068	−0.462
H(0) AP				0.745 *	0.639 *	0.278	0.568	−0.069	0.100	−0.177
H(0) ML					0.420	0.030	0.329	0.169	−0.272	−0.589
H(2) AP						0.320	0.674 *	0.032	0.480	0.134
H(2) ML							0.599	0.281	0.688 *	0.573
H(4.5) AP								−0.063	0.601	0.321
H(4.5) ML									0.348	0.189
SaEn AP										0.856 **

PM: Post-work; NPRS: Numeric Pain Rating Scale; H: Hurst Exponent; SaEn: Sample Entropy; AP: Anteroposterior; ML: Mediolateral; *: p<0.05; **: p<0.01.

**Table A9:** Correlation Matrix of Nonlinear Measurements on Day 3 During the AM Period and NPRS Sum (Day 3 AM).

	H(−5)		H(0)		H(2)		H(4.5)		SaEn	
	AP	ML	AP	ML	AP	ML	AP	ML	AP	ML
NPRS Sum	−0.631	−0.587	−0.812 **	−0.860 **	−0.330	−0.455	−0.786 **	−0.780 **	−0.451	−0.703 *
H(−5) AP		0.932 **	0.708 *	0.728 *	0.005	0.255	0.542	0.627	0.542	0.613
H(−5) ML			0.569	0.714 *	−0.060	0.409	0.575	0.661 *	0.513	0.490
H(0) AP				0.893 **	0.384	0.451	0.765 **	0.764 *	0.342	0.557
H(0) ML					0.281	0.553	0.770 **	0.885 **	0.454	0.500
H(2) AP						0.522	0.613	0.271	−0.454	0.137
H(2) ML							0.667 *	0.633 *	0.075	0.170
H(4.5) AP								0.778 **	0.072	0.475
H(4.5) ML									0.565	0.462
SaEn AP										0.608

AM: Pre-work; NPRS: Numeric Pain Rating Scale; H: Hurst Exponent; SaEn: Sample Entropy; AP: Anteroposterior; ML: Mediolateral; *: p<0.05; **: p<0.01.

**Table A10:** Correlation Matrix of Nonlinear Measurements on Day 3 During the PM Period and NPRS Sum (Day 3 PM).

	H(−5)		H(0)		H(2)		H(4.5)		SaEn	
	AP	ML	AP	ML	AP	ML	AP	ML	AP	ML
NPRS Sum	−0.195	0.008	−0.207	−0.019	−0.404	0.060	−0.190	0.368	0.260	0.103
H(−5) AP		−0.163	0.923 **	−0.002	0.528	−0.579	0.811 **	−0.207	−0.058	0.365
H(−5) ML			0.099	0.793 **	−0.302	0.179	−0.151	0.530	−0.308	−0.038
H(0) AP				0.219	0.593	−0.562	0.860 **	−0.046	−0.127	0.455
H(0) ML					0.078	0.273	−0.006	0.601	−0.712 *	−0.275
H(2) AP						−0.022	0.780 **	0.014	−0.369	0.285
H(2) ML							−0.257	0.478	−0.331	−0.149
H(4.5) AP								−0.074	−0.012	0.674 *
H(4.5) ML									−0.539	0.023
SaEn AP										0.325

PM: Post-work; NPRS: Numeric Pain Rating Scale; H: Hurst Exponent; SaEn: Sample Entropy; AP: Anteroposterior; ML: Mediolateral; *: p<0.05; **: p<0.01.

**Table A11:** Correlation Matrix of Nonlinear Measurements on Day 5 During the AM Period and NPRS Sum (Day 5 AM).

	H(−5)		H(0)		H(2)		H(4.5)		SaEN	
	AP	ML	AP	ML	AP	ML	AP	ML	AP	ML
NPRS Sum	−0.012	−0.046	0.129	−0.130	0.129	−0.415	−0.064	−0.308	0.240	0.457
H(−5) AP		0.738 *	0.846 **	0.724 *	0.589	−0.448	0.685 *	0.532	0.350	0.623
H(−5) ML			0.878 **	0.969 **	0.504	0.077	0.848 **	0.755 *	0.176	0.632
H(0) AP				0.888 **	0.550	−0.197	0.856 **	0.728 *	0.388	0.753 *
H(0) ML					0.406	0.085	0.810 **	0.819 **	0.223	0.593
H(2) AP						0.042	0.750 *	0.542	−0.021	0.314
H(2) ML							0.229	0.345	−0.418	−0.442
H(4.5) AP								0.801 **	0.071	0.457
H(4.5) ML									0.283	0.436
SaEn AP										0.769 **

AM: Pre-work; NPRS: Numeric Pain Rating Scale; H: Hurst Exponent; SaEn: Sample Entropy; AP: Anteroposterior; ML: Mediolateral; *: p<0.05; **: p<0.01.

**Table A12:** Correlation Matrix of Nonlinear Measurements on Day 5 During the PM Period and NPRS Sum (Day 5 PM).

	H(−5)		H(0)		H(2)		H(4.5)		SaEN	
	AP	ML	AP	ML	AP	ML	AP	ML	AP	ML
NPRS Sum	−0.276	−0.280	−0.215	−0.349	−0.722 *	−0.562	−0.317	−0.506	−0.071	−0.309
H(−5) AP		0.995 **	0.742 *	0.542	0.034	0.136	−0.507	0.132	−0.587	0.005
H(−5) ML			0.745 *	0.596	0.070	0.173	−0.530	0.144	−0.592	−0.017
H(0) AP				0.696 *	−0.004	0.445	−0.453	−0.027	−0.318	0.303
H(0) ML					0.326	0.663 *	−0.426	0.352	−0.333	0.161
H(2) AP						0.649 *	0.309	0.721 *	0.387	0.096
H(2) ML							−0.025	0.530	0.404	0.500
H(4.5) AP								−0.001	0.392	0.502
H(4.5) ML									0.330	−0.069
SaEn AP										0.245

PM: Post-work; NPRS: Numeric Pain Rating Scale; H: Hurst Exponent; SaEn: Sample Entropy; AP: Anteroposterior; ML: Mediolateral; *: p<0.05; **: p<0.01.
